# Idleness and Insanity in Prisons

**Published:** 1897-08

**Authors:** 


### Abstracts and Selections.

### Idleness and Insanity in Prisons.

The following press dispatch tells a tale that perhaps deserves some editorial comment from a medical point of view:

NEW YORK, July 22.—Since the anticonvict labor law went into operation on January 1, twenty prisoners in the Kings county penitentiary have lost their reason. Madness began to assert itself without anything to attract public attention. It attacked the victims one at a time until last week, when nine convicts, under the dull torture of enforced idleness, went mad. Five of these unfortunate men were removed yesterday from the penitentiary to the State asylum at Matteawan. The remaining four will probably be disposed of to-day. Three are federal prisoners, and their destination is the asylum at Washington. Madness in one case is laid at the door of mental torture, long sustained. In all the other cases enforced idleness—twenty hours of each day passed behind iron bars in trap-like cells—seems to give the explanation. The increase of lunacy as shown by figures among the male convicts is alarming. The records of 1890 show one case of lunacy; in 1891, three; in 1892, one; and for the three following years a total of four. Then prison work fell off, and for the first seven months of 1896, a period corresponding to the time during which the anticonvict labor law has been in operation, nineteen prisoners lost their reason.

The prophecy was made when a similar measure was being pressed prohibiting convict labor in another State than New York by the head of one of the chief penal institutions, that the result would be so large an increase of lunacy amongst the convicts that they might as well turn the prisons at once into asylums, only reserving a space for the necessary and inevitable preliminary lapse from reason to occur in. This was uttered as a rather forcible statement of the probable outcome of such a law as it appeared to him, but it is one that contained a large element of truth and only a moderate amount of exaggeration. Idleness and confinement would tax the resistance of an intellect that has abundant resources, and much more the untrained and more or less degenerate brain of the average prison inmate who has necessarily to be deprived to a large extent of the saving influences of companionship and association with his fellows.

From a purely philanthropic point of view it is not a justifiable piece of legislation to enact laws that deprive men of their reason, and it may be a question whether if tested it might not come under the constitutional prohibition of cruel and unusual punishments. In case of prisoners convicted before the passage of such acts, they constitute in spirit and in fact, if not in law, a most cruel and inhuman aggravation of the punishment to which they were sentenced.

There is an economic as well as a humane aspect in which the facts may be considered. Mental breakdowns under such conditions is not the most hopeful type of insanity, and it promises to largely increase the burden upon the community. A convict may reform, many of them do to a certain degree, and make harmless if not very valuable members of society. It is the aim of philanthropists to bring this about, and to make penitentiaries reformatories rather than places of vindictive punishment. If their function is to become manufactories of chronic dements it will be of very little advantage; the insanity added to the original evil tendencies will hardly make them any safer for the community, while it will insure their becoming dependents upon the taxpayers. In no way it can be viewed is the anti-convict labor legislation advantageous or respectable.

It is probable that the labor unions and others who have favored these acts gave very little attention to any possible consequences except the immediate results they had in view. It would not be creditable to them to suppose they would deliberately wish to condemn all convicts of all grades of guilt to a possible and even probable fate that is commonly described as almost or altogether worse than death, however true this may be, and yet this is just what they have practically done. It is useless to say that they can be employed without competition with outside labor, for if that were possible it would have been resorted to in the New York institution—it must be presumed that it was either impracticable or was made so by the wording of the law; no prison authorities would readily abandon their best resource for government as well as for profit in such institutions.

This is only one of the many instances where inconsiderate law-making is done that ought to have been modified or avoided. If enlightened medical counsels could prevail, if medical men could take part in making our laws, or if something besides what is called practical politics could be more considered by our legislators, such a disgrace to the State as this inhuman and backward step in prison administration would have probably not occurred. It is not at all necessary that prison labor should injuriously compete with workers outside, but the outcry at the present time is too indiscriminating, and inconsiderate legislation is too often demanded with such results as are shown in the paragraph quoted.—*Editorial in Journal of the American Medical Association.*

